# T-cell costimulation blockade is effective in experimental digestive and lung tissue fibrosis

**DOI:** 10.1186/s13075-018-1694-9

**Published:** 2018-08-29

**Authors:** Gonçalo Boleto, Christophe Guignabert, Sonia Pezet, Anne Cauvet, Jérémy Sadoine, Ly Tu, Carole Nicco, Camille Gobeaux, Frédéric Batteux, Yannick Allanore, Jérôme Avouac

**Affiliations:** 1 Université Paris Descartes, Sorbonne Paris Cité, INSERM U1016, Institut Cochin, CNRS UMR8104, Paris, France; 2 Université Paris Descartes, Sorbonne Paris Cité, Service de Rhumatologie A, Hôpital Cochin, 27 rue du Faubourg Saint Jacques, 75014 Paris, France; 3 INSERM UMR_S 999, Le Plessis-Robinson, France; 4 Université Paris-Sud, Université Paris-Saclay, Le Kremlin-Bicêtre, France; 5 EA 2496 Pathologie, Imagerie et Biothérapies Orofaciales, UFR Odontologie, Université Paris Descartes and PIDV, PRES Sorbonne Paris Cité, Montrouge, France; 6 Clinical Chemistry Laboratory, Cochin and Hôtel-Dieu Hospitals, Paris, France

**Keywords:** Pulmonary fibrosis, Pulmonary hypertension, Gastrointestinal tract involvement, Systemic sclerosis, Abatacept

## Abstract

**Background:**

We aimed to investigate the efficacy of abatacept in preclinical mouse models of digestive involvement, pulmonary fibrosis, and related pulmonary hypertension (PH), mimicking internal organ involvement in systemic sclerosis (SSc).

**Methods:**

Abatacept has been evaluated in the chronic graft-versus-host disease (cGvHD) mouse model (abatacept 1 mg/mL for 6 weeks), characterized by liver and intestinal fibrosis and in the Fra-2 mouse model (1 mg/mL or 10 mg/mL for 4 weeks), characterized by interstitial lung disease (ILD) and pulmonary vascular remodeling leading to PH.

**Results:**

In the cGvHD model, abatacept significantly decreased liver transaminase levels and markedly improved colon inflammation. In the Fra-2 model, abatacept alleviated ILD, with a significant reduction in lung density on chest microcomputed tomography (CT), fibrosis histological score, and lung biochemical markers. Moreover, abatacept reversed PH in Fra-2 mice by improving vessel remodeling and related cardiac hemodynamic impairment. Abatacept significantly reduced fibrogenic marker levels, T-cell proliferation, and M1/M2 macrophage infiltration in lesional lungs of Fra-2 mice.

**Conclusion:**

Abatacept improves digestive involvement, prevents lung fibrosis, and attenuates PH. These findings suggest that abatacept might be an appealing therapeutic approach beyond skin fibrosis for organ involvement in SSc.

**Electronic supplementary material:**

The online version of this article (10.1186/s13075-018-1694-9) contains supplementary material, which is available to authorized users.

## Background

Systemic sclerosis (SSc) is a rare life-threatening condition of autoimmune origin [1] defined by pathological fibrosis of the skin and internal organs [2]. T cells represent a major component of the infiltrate in the early inflammatory stage of the disease, exhibiting an activated phenotype with CD4^+^ T cells predominating over CD8^+^ T cells [3].

Cytotoxic T lymphocyte-associated molecule (CTLA)-4 is an immunoregulatory membrane receptor resulting in the downregulation of T-cell responses by inhibiting the costimulatory interactions of CD28-B7 [4]. We have recently shown that abatacept both prevents and induces regression of inflammation-driven dermal fibrosis in two complementary mouse models of SSc [5]. Preliminary clinical data have suggested benefits of abatacept on inflammatory joint involvement [6] and skin fibrosis [7] in SSc patients, and a randomized controlled trial is ongoing (NCT02161406). However, SSc prognosis depends on major organ involvement. Pulmonary complications including interstitial lung disease (ILD) and pulmonary hypertension (PH) remain the largest causes of mortality in SSc [1]. Digestive involvement is very frequent in SSc patients and can also lead to severe malabsorption.

We hypothesized that, beyond skin effects, abatacept may be efficient for the treatment of organ damage, in particular digestive, lung, and vessel fibrosis. Our aim was to assess the effects of abatacept in mouse models mimicking severe organ damage characterizing SSc.

## Methods

### Animals

Thirteen-week-old male and female C57BL6/J mice and 6-week-old female BALB/c mice were purchased from Janvier Laboratory (Le Genest Saint Isle, France). Thirteen-week-old male and female Fra-2 transgenic mice (SA5446 D-H3/FRA-2 (Tg4)) were obtained from a collaboration established with Sanofi Genzyme [8, 9]. B10.D2-enhanced green fluorescent protein (eGFP) mice were provided by Colette Kanellopoulos-Langevin, CDTA–CNRS–Orléans, France. All mice were housed in ventilated cages with sterile food and water ad libitum. All animals were treated in accordance with the Guide for the Care and Use of Laboratory Animals as adopted by INSERM, and approval was granted by the Ethics Committee of our University.

### Pharmacological treatment

Abatacept (CTLA-4-Ig) was provided by Bristol-Myers Squibb, and purified human IgG1 (MP Biomedicals, Illkirch, France) was used as a negative control. Abatacept at two different concentrations (1 mg/mL and 10 mg/mL) and control IgG1 were dissolved in 0.9% NaCl and injected every other day intraperitoneally at a dose of 100 μg/mouse, as used previously [10]. A dose of 1 mg/mL was used in the chronic graft-versus-host disease (cGvHD) model since it has been shown to be effective in this setting in a previous report [5]. To assess a potential dose effect of abatacept, two different dose regimens were used in the Fra-2 transgenic mouse model. The dosage of the drug was not adjusted to body weight. Both doses of abatacept, 1 mg/mL (3 mg/kg) and 10 mg/mL (30 mg/kg) were physiological since abatacept has been administered in preclinical studies up to 50 mg/kg in rodents without any sign of toxicity.

In the cGvHD model, treatment with abatacept or IgG1 started 5 days after transplantation and the outcome was analyzed after 6 weeks.

In Fra-2 mice, treatment started at the age of 13 weeks and the outcome was analyzed after 4 weeks.

### Murine sclerodermatous cGvHD

cGvHD was induced in BALB/c mice (H-2 d) by grafting allogeneic transplantation of 1 × 10^6^ bone marrow cells and 2 × 10^6^ splenocytes from 7-week-old to 8-week-old male B10.D2-eGFP mice (H-2 d), as previously described [5, 11, 12]. Recipient mice develop an inflammation-driven fibrosis resembling the early inflammatory stages of SSc which is responsible for liver and gastrointestinal tract damage. Intraperitoneal injections of abatacept 1 mg/mL (*n* = 12 mice) or control IgG1 (*n* = 12 mice) were performed every other day, starting 5 days after transplantation, which corresponds to a preventative setting. The outcome was analyzed after 6 week. Mice undergoing syngeneic transplantation of bone marrow cells and splenocytes served as controls (*n* = 8).

### Serum alanine aminotransferase and aspartate aminotransferase activities in the cGvHD model

Serum activity of alanine aminotransferase (ALT) and aspartate aminotransferase (AST) were used as markers of hepatocyte cytolysis. AST and ALT activities were quantified using a standard clinical automatic analyzer (Modular PP, Roche Diagnostics, Meylan, France).

### Histopathologic assessment of colon involvement in the cGvHD model

Fixed colon biopsies were embedded in paraffin. A 5-mm thick tissue section was stained with hematoxylin and eosin [13]. Slides were examined by standard bright-field microscopy (Nikon Eclipse 80i, Tokyo, Japan). The severity of colon involvement was semiquantitatively assessed on a scale of 0–4 according to the method described by Blazar et al. [14] by two examiners (GB and SP) blinded to the genotype and the treatment. The grading criteria were as follows: grade 0, normal; grade 0.5, occasional necrotic crypt cell, minimal infiltration in lamina propria and submucosa; grade 1, necrotic cells in up to 15% of crypts, minor infiltration of up to 20% of lamina propria (1- to 2-cell thickness in intermucosal areas and submucosa); grade 1.5, necrotic cells in up to 15% of crypts, minor infiltration of less than or equal to one-third of the lamina propria (1- to 2-cell thickness in intermucosal areas and submucosa); grade 2, necrotic cells in ≤ 25% of crypts, infiltration of less than or equal to one-third of the lamina propria (3-cell thickness in intermucosal areas and submucosa); grade 2.5, necrotic cells in 25% to 50% of crypts, infiltration of less than or equal to one-third of lamina propria (3- to 4-cell thickness in intermucosal areas and submucosa); grade 3, necrotic cells in greater than 50% of crypts, infiltration of lamina propria (5- to 6-cell thickness in intermucosal areas and submucosa) with loss of ≤ 25% of goblet cells; grade 3.5, necrotic cells in greater than 50% of crypts, infiltration of lamina propria resulting in displacement of ≤ 50% of mucosa with loss of 50% of goblet cells; and grade 4, necrotic cells in greater than 50% of crypts, infiltration of lamina propria resulting in displacement of greater than 50% of mucosa with loss of 75% to 100% of goblet cells.

### Effects of abatacept in the Fra-2 model

Transgenic mice expressing the fra-2 gene under control of the ubiquitous major histocompatibility complex class I antigen H2Kb promoter display systemic fibrosis, microangiopathy, and PH. These manifestations follow a similar temporal sequence as seen in human SSc. The presence of typical capillary changes, pulmonary fibrosis, and PH is unique among murine models for SSc. A significant decrease in capillary density occurs from weeks 12–13 [15]. In the lungs, obliteration of pulmonary arteries, accompanied by perivascular inflammatory infiltrates, becoming apparent at an age of 12–13 weeks precedes the onset of fibrosis by 2–3 weeks [16]. Regarding the occurrence of PH, Fra-2 transgenic mice develop severe vascular remodeling of pulmonary arteries resembling human SSc-PH. Histological features typical for SSc-PH, such as intimal thickening with concentric laminar lesions, medial hypertrophy, perivascular inflammatory infiltrates, and adventitial fibrosis, are frequently detected [9, 17].

Three groups of Fra-2 transgenic mice and one group of C57BL/6 mice were treated by intraperitoneal injections of abatacept 1 mg/mL (*n* = 8 Fra-2 mice), abatacept 10 mg/mL (*n* = 8 Fra-2 mice), or control IgG1 (*n* = 6 Fra-2 mice and 5 C57BL/6 mice) every other day, starting at the age of 13 weeks. Injections were performed for 4 weeks. Mice were killed by cervical dislocation at the age of 17 weeks.

It is important to note that, at week 13, lung fibrosis is not present in this model [18], which means that our therapeutic approach for this outcome was preventative. On the other hand, obliteration of pulmonary arteries is usually detected at week 12 in Fra-2 mice [15, 16], which supports a curative approach of PH.

### Assessment of fibrosing alveolitis by chest microcomputed tomography in the Fra-2 model

Fibrosing alveolitis of mice was evaluated using microcomputed tomography (microCT) 2 days before sacrifice, as previously reported [19]. CT images were obtained with a Perkin Elmer’s Quantum FX system (Caliper Life Sciences). The animals were placed in the supine position on the CT table. Mice were sedated with 3–4% isoflurane anesthesia at 0.5–1.5 L/min for induction by a nose cone. Anesthesia was maintained with 2.5–3% isoflurane at 400–800 mL/min during the acquisition. During image acquisition, thoracic breathing movements were recorded, detecting the up- and downward movement of the thorax. Images were acquired throughout the spontaneous respiratory cycle. Only images acquired during expiration were analyzed. Images were acquired with the following parameters: 90 kV x-ray source voltage, 160 μA current. Total scanning time was approximately 4.5 min per mouse choosing from the list mode made by the constructor with the gating parameter. Tomograms were reconstructed using Rigaku software. The analysis starts with the isolation of lung tissue by manually drawing a volume of interest. Analysis of lung density and drawing was performed with Ctan Brucker software. Lung density was measured in Hounsfield Units (HU) after calibration. A phantom calibration was made on the acquisition Rigaku software: a water-filled 1.5-mL tube inside a 2-mL tube was scanned. Based on full-stack histograms of a manually delimited volume of interest containing only water or air, the mean grayscale index of water was set at 0 HU, and the grayscale index of air was set at −1000 HU. This value was reported in the Ctan Brucker software. Means of lung density of both groups were achieved by evaluation of all CT scans acquired from the apices to the bases of the lungs. Furthermore, the volume of functional lung parenchyma corresponding to functional residual capacity (FRC) was manually drawn by excluding the fibrosis area and vessels. Percentages of FRC on total lung volumes were calculated. The CT expert (JS) was blinded to the background of the mice, to the treatment, and to the results of the histological assessment.

### Histopathologic assessment of fibrosing alveolitis in the Fra-2 model

Paraffin-embedded lung sections (5 μm) were stained with hematoxylin and eosin. The severity of fibrosing alveolitis was semiquantitatively assessed according to the method described by Ashcroft et al. by two examiners blinded to the genotype and the treatment (SP and AC) [19, 20]. Lung fibrosis was graded on a scale of 0 to 8 by examining randomly chosen fields of the left upper lobe. The grading criteria were as follows: grade 0, normal lung; grade 1, minimal fibrous thickening of alveolar walls; grade 3, moderate thickening of walls without obvious damage; grade 5, increased fibrosis with definite damage and formation of fibrous bands; grade 7, severe distortion of structure and large fibrous areas; and grade 8, total fibrous obliteration. Grades 2, 4, and 6 were used as intermediate stages between these criteria. This analysis was performed two examiners (SP and AC) in blinded manner. All images were taken with a Lamina multilabel slide scanner.

### Collagen measurements in Fra-2 transgenic mice

The collagen content in lesional lung samples, taken from the same lobe for each mouse, was explored by hydroxyproline assay, as previously described [5, 9]. Briefly, each sample was hydrolyzed and titrated to a pH of 7. This solution was combined with chloramine T and p-dimethylaminobenzaldehyde in perchloric acid and read at 557 nm with a spectrophotometer (Molecular Devices, Sunnyvale, CA). This experiment was performed for two samples per mouse analyzed.

### Nonlinear microscopy and second harmonic generation processing in Fra-2 transgenic mice

A multiphoton inverted stand Leica SP5 microscope (Leica Microsystems Gmbh, Wetzlar, Germany) was used for tissue imaging. A Ti:Sapphire Chameleon Ultra (Coherent, Saclay, France) with a center wavelength at 810 nm was used as the laser source for second harmonic generation (SHG) and two-photon excited fluorescence (TPEF) signals. The laser beam was circularly polarized to ensure isotropic excitation of the sample regardless of the orientation of fibrillar collagen. A Leica Microsystems HCX IRAPO 25×/0.95 W objective was used to excite and collect SHG and TPEF. Signals were detected in epi-collection through a 405/15-nm and a 525/50 bandpass filter, respectively, by NDD PMT detectors (Leica Microsystems) with a constant voltage supply, at constant laser excitation power, allowing direct comparison of SHG intensity values. Two fixed thresholds were chosen to distinguish biological material from the background signal (TPEF images) and specific collagen fibers (SHG images). SHG score was established by comparing the area occupied by the collagen relative to the sample surface. Image processing and analysis (thresholding and SHG scoring) were performed using ImageJ homemade routines (https://imagej.nih.gov/ij/) as previously described [8]. Results were normalized to control C57/BL6 mice.

### Lung biomarker measurement in Fra-2 transgenic mice

Selected fibrogenic markers were quantified by enzyme-linked immunosorbent assay (ELISA) in lesional lungs of Fra-2 transgenic mice. Proteins were extracted from lesional lungs, taken from the same specific lobe for each mouse, with Tissue Protein Extraction Reagent (T-PER®; Thermo Fischer Scientific, Villebon Sur Yvette, France) with Halt Protease Inhibitor Single-Use Cocktail, EDTA-free (Thermo Fischer Scientific). Total proteins were dosed by the BCA technique. All proteins were quantified in lesional lungs with mouse ELISA kits according to the instructions of the manufacturers. The detection limit was > 10 pg/mL. The following markers were assayed based on their relevance in the pathogenesis of SSc and animal models of SSc [2, 21–23] and on previous experience with these markers in the Fra-2 mouse model [19]: transforming growth factor (TGF)-β1, osteopontin (OPN), monocyte chemoattractant protein (MCP)-1, and TIMP metallopeptidase inhibitor-1 (TIMP-1) (all from R&D systems, Lille, France). Results are expressed as amount of biomarker per mg total tissue protein.

### Immunostaining of lesional lung sections

Immunofluorescence and immunohistochemistry were performed after antigen retrieval and blocking with 2% bovine serum albumin (BSA) for 1 h at room temperature.

Type 1 (M1) and type 2 (M2) macrophages were detected by immunofluorescence in lesional lung sections. To detect M1 macrophages, sections were incubated with goat inducible nitric oxide synthase (iNOS) antibodies (Invitrogen, Paris, France; dilution 1:200), monoclonal CD11c antibodies (Abcam, Cambridge, UK; dilution 1:20), and monoclonal F4/80 antibodies (Abcam; dilution 1:100) overnight at 4 °C. To detect M2 macrophages, lesional lung sections were incubated with polyclonal cMAF antibodies (Santa Cruz, San Diego, CA; dilution 1:50), polyclonal arginase antibodies (Santa Cruz; dilution 1:200), and F4/80 [24]. Additional stainings by immunofluorescence included Ki-67 (polyclonal antibodies, dilution 1:550; Abcam) and CD3 (monoclonal antibodies, dilution 1:50; Abcam) to identify proliferative T cells. The following secondary antibodies were used for 1 h at room temperature [25]: Alexa-Fluor-labeled donkey anti-rabbit 488 (1:200), donkey anti-goat 594 (1:100), chicken anti-rat 647 (1:200), and goat anti-hamster 594 (1:100) (all from Life Technologies, Darmstadt, Germany). All sections were counterstained with DAPI.

We also performed immunohistochemistry in lesional colon sections for CD45 (polyclonal antibodies, dilution 1:200; Novus, Lille, France) to quantify inflammatory cells and Annexin-V (polyclonal antibodies, 1:100; Abcam) to detect apoptotic/necrotic cells. Polyclonal horseradish peroxidase-labeled goat anti-rabbit immunoglobulins were used as secondary antibodies (1:200; Agilent Technologies, Les Ulis, France). Staining was visualized with DAB peroxidase substrate solution (Sigma-Aldrich, Darmstadt, Germany).

All images were captured with a Lamina multilabel slide scanner (PerkinElmer, Villebon Sur Yvette, France). Cell quantification was performed by two independent investigators (GB and SP) according an automated counting method using the ImageJ software.

### Hemodynamic measurements and assessment of vessel remodeling in Fra-2 transgenic mice

Right ventricular systolic pressure (RVSP) and heart rate were determined in unventilated mice under isoflurane anesthesia (1.5–2.5%, 2 L O_2_/min) using a closed chest technique, by introducing a catheter (1.4-F catheter; Millar Instruments Inc., Houston, TX) into the jugular vein and directing it to the right ventricle [26–28]. After all the hemodynamic assessments were completed, blood was collected by direct cardiac puncture and sacrificed by exsanguination. The heart and lungs were then removed en bloc and right ventricular hypertrophy (RVH) was determined by the Fulton index measurement (right ventricle/left ventricle plus septum (RV/LV + S)) [26–28]. The pulmonary circulation was flushed with 5 mL buffered saline at 37 °C, and then the left lung was prepared for morphometric analyses and the right lung was quickly harvested, immediately snap-frozen in liquid nitrogen and kept at −80 °C.

Morphometric analyses were performed on paraffin-embedded lung sections stained using hematoxylin and eosin and alpha smooth muscle actin (α-SMA). α-SMA was detected by incubation with monoclonal anti-α-SMA antibody (clone 1A4; Dako Glostrup, Denmark) at a dilution of 1:100 overnight at 4 °C. A Vectastain ABC kit was the used according to the manufacturer’s instructions (Vector Laboratories, Burlingame, CA) and slides were then counterstained with hematoxylin (Sigma-Aldrich). The percentage of wall thickness ((2 × medial wall thickness/external diameter) × 100) and of muscularized vessels were determined as previously described [29]. All morphometric analyses were performed by one observer (CG) blinded to genotype and treatment conditions.

### Statistics

All data are expressed as median values ± interquartile range (IQR). Multiple group comparisons were analyzed using a post-hoc Dunnett’s test. The Mann-Whitney *U* test was used for a two-group comparison. *P* < 0.05 (all two-sided) was considered significant.

## Results

### Abatacept alleviates liver cytolysis and gut involvement in experimental cGvHD

**Fig. 1:**
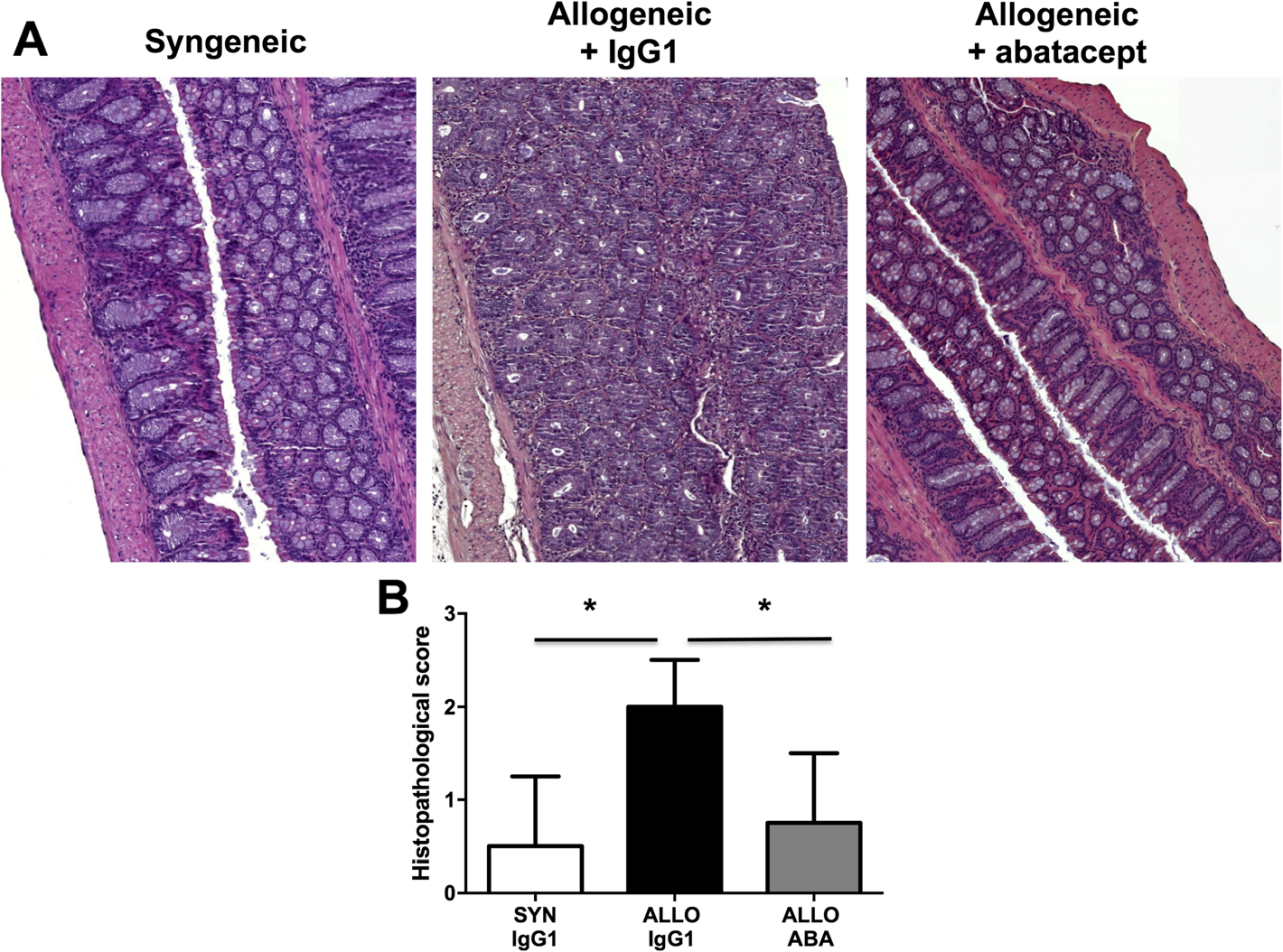
Abatacept prevents cGvHD-associated colon involvement. **a** Representative 5-mm thick colon sections stained by hematoxylin and eosin showing syngeneic BALB/c mice and cGvHD mice treated by control IgG1 or abatacept 1 mg/mL. Submucosal infiltration by mononuclear cells and destruction of crypts in abatacept-treated cGvHD mice are decreased when compared with IgG1-treated cGvHD mice. **b** Histological score of colon involvement decreased significantly upon treatment with abatacept 1 mg/mL in cGvHD mice compared with IgG1-treated cGvHD mice. A total of 32 mice were used (12 allogeneic (ALLO) control IgG1-treated mice, 12 abatacept (ABA) 1 mg/mL-treated mice, and 8 control syngeneic (SYN) BALB/c mice). Values are the median ± IQR. Statistics are from post-hoc Dunnett’s multiple comparison test. *P < 0.05

Treatment of allogeneic mice with abatacept led to a significant reduction in ALT (24%, *P* = 0.014) and AST levels (61%, *P* < 0.001) compared with IgG1-treated allogeneic mice (Additional file 1A, B). Pathological analysis of the colon revealed reduced inflammatory cell infiltration in the lamina propria, necrotic crypt cells, and loss of goblet cells in allogeneic abatacept-treated mice compared with IgG1-treated mice (Fig. 1a). A significant 47% reduction in the histological score, evaluating inflammatory change, was observed in allogeneic mice treated with abatacept (*P* = 0.019) (Fig. 1b). Consistent with this observation, submucosal CD45^+^ inflammatory cell infiltration and the number of annexin V-positive dead cells were markedly reduced in allogeneic abatacept-treated mice (Additional file 2A, B).

### Abatacept alleviates lung fibrosis in the Fra-2 mouse model

**Fig. 2:**
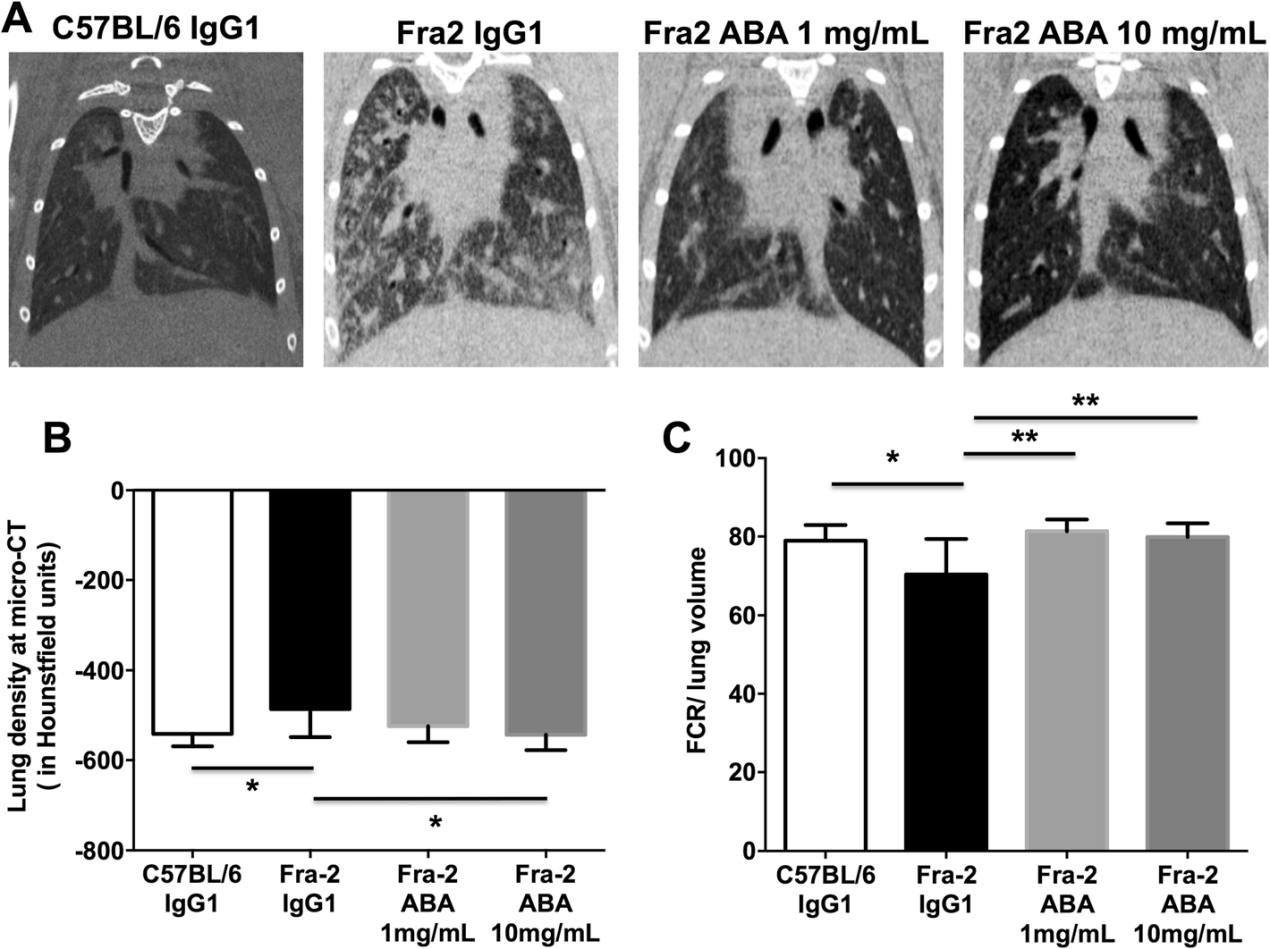
Abatacept protects against fibrosing alveolitis in the Fra-2 mouse model. Evaluation by CT-scan. **a** Treatment with abatacept (ABA) prevents lung fibrosis in Fra-2 transgenic mice; representative pictures of microcomputed tomography. **b** Decreased lung density at microcomputed tomography (micro-CT) in Fra-2 transgenic mice treated with abatacept 10 mg/mL compared with control IgG1-treated mice. **c** Reduced residual lung volume, expressed as the percentage of functional residual capacity (FRC) on total lung volume in Fra-2 transgenic mice treated with abatacept 1 mg/mL and 10 mg/mL compared with control IgG1-treated mice. A total of 27 mice were used (5 C57BL/6 mice, 6 Fra-2 control IgG1, 8 Fra-2 abatacept 1 mg/mL, and 8 Fra-2 abatacept 10 mg/mL). Values are the median ± IQR. Statistics are from post-hoc Dunnett’s multiple comparison test. *P < 0.05, **P < 0.01

Mice treated with abatacept 10 mg/mL showed decreased lung density to levels similar to control C57BL/6 mice when assessed by chest microCT (Fig. 2a, b). The FRC significantly improved in both groups of abatacept-treated mice, with similar values to control C57BL/6 mice (Fig. 2c).

**Fig. 3:**
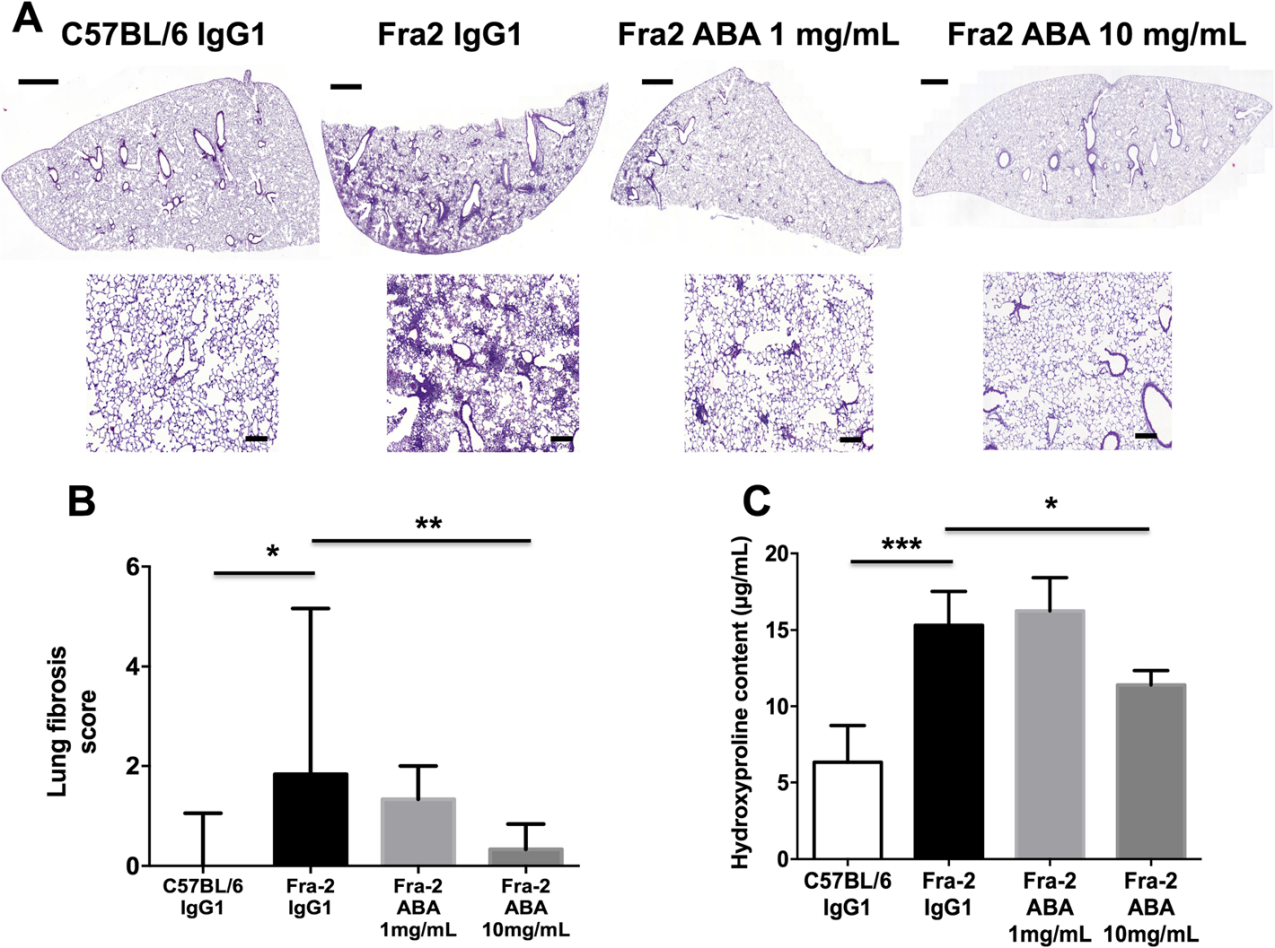
Abatacept 10 mg/mL prevents lung fibrosis in Fra-2 transgenic mice. Evaluation by histology. **a** Treatment with abatacept (ABA) 10 mg/mL prevents lung fibrosis in Fra-2 transgenic mice; representative lung sections stained by hematoxylin and eosin. Scale bars = 100 μm. **b** Histological lung fibrosis score decreased significantly on treatment with abatacept 10 mg/mL compared with mice receiving abatacept 1 mg/mL and control IgG1-treated mice. **c** Hydroxyproline content in lesional lungs of Fra-2 mice markedly decreased on treatment with abatacept 10 mg/mL compared with mice receiving abatacept 1 mg/mL and control IgG1-treated mice. A total of 27 mice were used (5 C57BL/6 mice, 6 Fra-2 control IgG1, 8 Fra-2 abatacept 1 mg/mL, and 8 Fra-2 abatacept 10 mg/mL). Values are the median ± IQR. Statistics are from post-hoc Dunnett’s multiple comparison test. *P < 0.05, **P < 0.01, ***P < 0.001

Lung specimens from IgG1-treated mice exhibited features of fibrosing alveolitis (Fig. 3a). On treatment with abatacept, a significant 79% reduction of the lung fibrosis score was observed at a dose of 10 mg/mL compared with mice treated with IgG1 (*P* = 0.009) (Fig. 3a, b). Consistent with CT and histological analysis, hydroxyproline content was also reduced by 31% in lung specimens from mice treated with abatacept 10 mg/mL (*P* = 0.044) (Fig. 3c).

SHG showed a preferential perivascular distribution of fibrosis in IgG1-treated mice, which was consistent with fibrosing alveolitis (Additional file 3A). Scoring of fibrillar collagen deposits confirmed a significant decrease in collagen scoring in Fra-2 mice receiving abatacept 10 mg/mL compared with Fra-2 mice treated with IgG1 (Additional file 3B).

Treatment with abatacept 10 mg/mL markedly reduced lung protein levels of MCP1 by 79% (*P* = 0.043), OPN by 87% (*P* = 0.039), and TGF-β by 69% (*P* = 0.013). Levels of TGF-β were also reduced by 61% on treatment with abatacept 1 mg/mL (*P* = 0.037) (Additional file 4A–D).

### Abatacept reverses PH in the Fra-2 mouse model

**Fig. 4:**
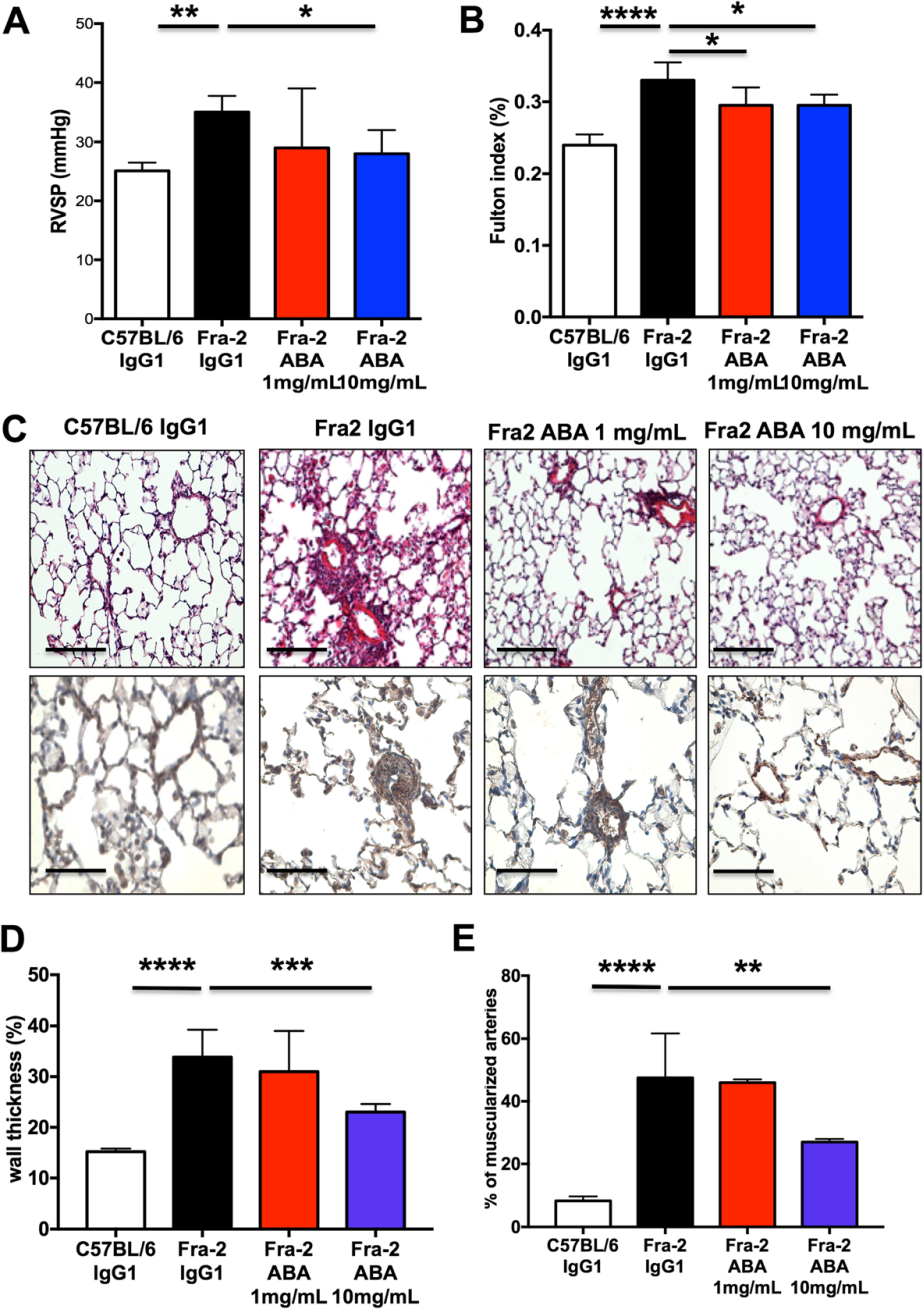
Abatacept alleviates pulmonary hypertension in Fra-2 transgenic mice. **a** Right ventricular systolic pressure (RVSP) and **b** right ventricular hypertrophy assessed by the Fulton index. **c** Representative images of hematoxylin and eosin staining (upper row) and representative images of α-smooth muscle actin (α-SMA) immunohistostaining (lower row) showing a substantial reduction in the percentage of medial wall thickness (**d**) and a significant reduction in the percentage of distal artery muscularization (**e**) in Fra-2 mice treated with abatacept (ABA) 1 mg/mL and 10 mg/mL compared with control IgG1-treated mice. Scale bars = 100 μm. A total of 27 mice were used (5 C57BL/6 mice, 6 Fra-2 control IgG1, 8 Fra-2 abatacept 1 mg/mL, and 8 Fra-2 abatacept 10 mg/mL). Values are the median ± IQR. Statistics are from post-hoc Dunnett’s multiple comparison test. *P < 0.05, **P < 0.01, ***P < 0.001, ****P < 0.0001

On treatment with abatacept 10 mg/mL, a substantial reduction of RVSP (28.1 ± 1.5 mmHg vs. 36.0 ± 5.1 mmHg, *P* = 0.037) was observed compared with IgG1-treated mice (Fig. 4a). RVH was also significantly decreased with abatacept 1 mg/mL (0.29 ± 0.01% vs. 0.33 ± 0.01%, *P* = 0.037) and 10 mg/mL (0.29 ± 0.01% vs. 0.33 ± 0.01%, *P* = 0.037) (Fig. 4b). Likewise, abatacept 1 mg/mL and abatacept 10 mg/mL were associated with a significant decrease in percentage medial wall thickness (Fig. 4c, d) and number of muscularized distal pulmonary arteries (Fig. 4c, e).

### Abatacept reduces T-cell proliferation and M1/M2 macrophage infiltration in lesional lungs

**Fig. 5:**
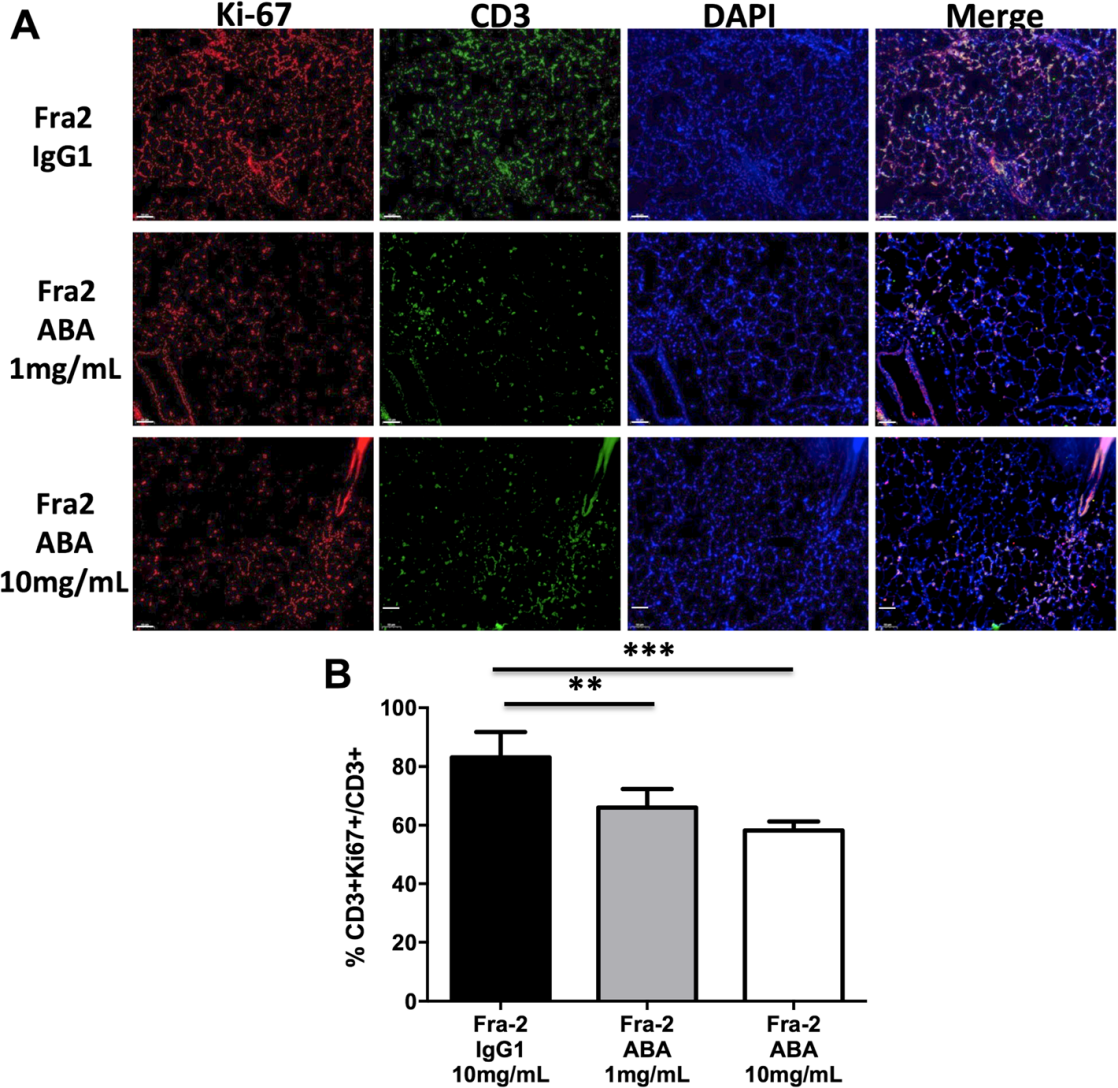
Abatacept reduces T-cell proliferation in lesional lungs of Fra-2 transgenic mice. **a** Representative lung sections stained by the proliferation marker Ki-67 and CD3 to quantify the number of proliferative T cells. **b** Abatacept (ABA) significantly reduced the number of proliferative CD3^+^Ki-67^+^ cells reported on the total number of CD3^+^ cells. A total of 22 mice were used (6 Fra-2 control IgG1, 8 Fra-2 abatacept 1 mg/mL, and 8 Fra-2 abatacept 10 mg/mL). Values the median ± IQR. Statistics are from post-hoc Dunnett’s multiple comparison test. **P < 0.01, ***P < 0.001

To evaluate the effects of abatacept on T-cell proliferation, we detected the expression of the proliferation marker Ki-67 in lesional lungs (Fig. 5a). The ratio of T cells expressing Ki-67 to total CD3-positive T cells was found significantly decreased by 21% (*P* = 0.009) and 29% (*P* = 0.001) in mice treated with abatacept 1 mg/mL and 10 mg/mL, respectively, compared with mice receiving control IgG1 (Fig. 5a, b).

**Fig. 6:**
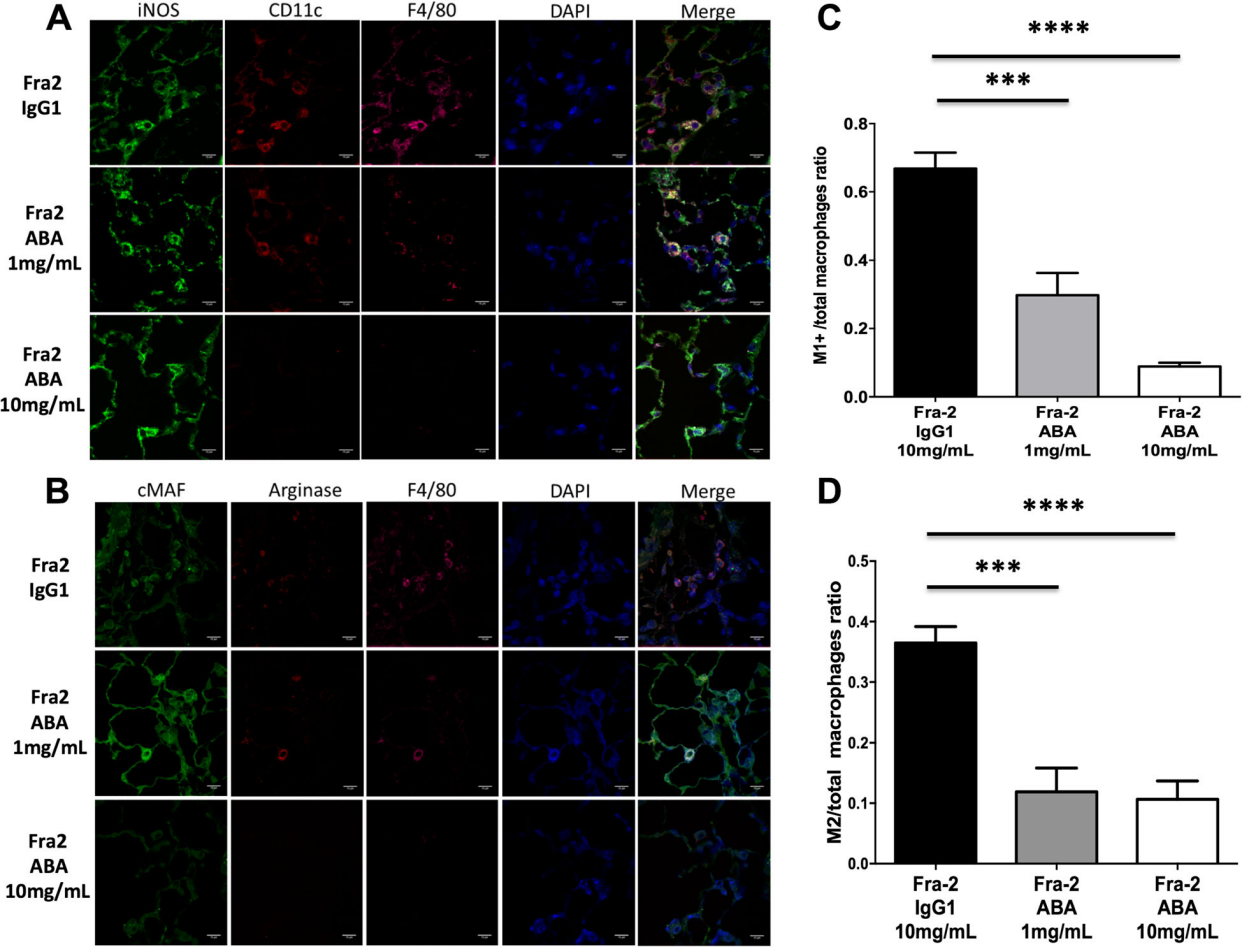
Abatacept reduces T-cell proliferation and M1/M2 macrophage infiltration in lesional lungs of Fra-2 transgenic mice. **a** Representative lung sections stained for iNOS, CD11c, and F4/80 to detect type 1 (M1) macrophages, and **b** stained for cMAF, arginase, and F4/80 to detect type 2 (M2) macrophages. Abatacept (ABA) 1 mg/mL and 10 mg/mL significantly reduced the number of **c** M1 and **d** M2 macrophages. A total of 22 mice were used (6 Fra-2 control IgG1, 8 Fra-2 abatacept 1 mg/mL, and 8 Fra-2 abatacept 10 mg/mL). Values are the median ± IQR. Statistics are from post-hoc Dunnett’s multiple comparison test. ***P < 0.001, ****P < 0.0001

To evaluate whether abatacept influences the outcome of Fra-2 mice by regulating infiltration of M1/M2 macrophages, we quantified the number of proinflammatory M1 macrophages and profibrotic M2 macrophages in lesional lungs (Fig. 6a, b). The ratio of M1 and M2 macrophages to total macrophages dramatically decreased in mice treated with abatacept 1 mg/mL (by 72%, *P* < 0.001, and 93%, *P* < 0.001, respectively) and 10 mg/mL (by 58%, *P* = 0.003, and 70%, *P* < 0.001, respectively) compared with those injected with control IgG (Fig. 6c, d).

## Discussion

In this study, we demonstrate how the inhibition of T-cell costimulation reduces the severity of SSc organ damage in two complementary experimental models. Abatacept was well tolerated in both mouse models with no weight loss during the whole treatment period (control IgG1 +1.50 ± 1.12 g, abatacept 1 mg/mL +1.13 ± 1.35 g, and abatacept 10 mg/mL +0.97 ± 1.07 g).

Abatacept prevents liver and colon damage in the cGvHD model. As in the early stages of human SSc, this model is characterized by dense inflammatory infiltrates in affected organs (skin, liver, gastrointestinal tract), composed of mononuclear cells, T cells, and monocyte-macrophages, which contribute to the initial activation of resident fibroblasts by the release of profibrotic mediators. However, the degree of persistent cellular infiltrate is rather more than is typical of human SSc [30, 31].

Fra-2 transgenic mice are characterized by features of human vasculopathy, including PH, paralleled by fibrosing alveolitis similar to that seen in patients with SSc. Abatacept substantially improves parameters evaluating both interstitial lung inflammation and fibrosis in this mouse model, supporting the concept that the beneficial effects of abatacept are not only restricted to inflammatory changes. Abatacept also markedly alleviates vascular remodeling and signs of PH.

These results extend previous findings observed in experimental models of inflammation-driven fibrosis [5, 32, 33] and hypersensitivity pneumonitis [34]. Targeting T cell-mediated responses made it possible to interfere with tissue remodeling leading to lung fibrosis and PH in the Fra-2 model [18], in line with the attenuation, upon T-cell costimulation blockade, of cardiac hypertrophy and fibrosis that were observed in experimental models of systemic hypertension and pathological cardiac hypertrophy [35, 36].

It is noteworthy that, despite the efficacy of abatacept 1 mg/mL in the cGvHD model and the positive signal observed on several parameters with this dose in Fra-2 mice, significant improvements in the Fra-2 model were mostly reached with the dose of 10 mg/mL. The contribution of T cells for disease phenotype may partly explain this result. In the cGvHD, the role of T cells is preponderant [37]. In Fra-2 transgenic mice, although inflammatory infiltrates seem to contribute to disease pathogenesis, their implication seems less fundamental, since reciprocal bone marrow reconstitution experiments and the assessment of Fra-2 mice lacking functional B and T lymphocytes showed that T and B cells are not essential for the pathogenesis of pulmonary fibrosis [18].

It is difficult to speculate on organ-specific differences in treatment response with abatacept, which depends on the dose received and the mouse model. The dose of 1 mg/mL seems relevant to treat inflammation-driven skin fibrosis given the positive results observed with this dose in bleomycin-induced dermal fibrosis and cGvHD [5]. Higher doses appear necessary to treat more severe organ complications such as ILD and PH.

The benefit conferred by abatacept treatment may be that it targets T-cell costimulation and thus their optimal activation. Furthermore, targeting costimulation requires the targeting of CD80/CD86-bearing macrophages and B cells, which contributes to the therapeutic effect, affecting T cell-associated B cell and macrophage responses [38]. Fibrosis formation requires the combined action of Th2 cells and innate immune cells. In the Fra-2 mouse model, we identified an intense M1-polarized innate response, which we speculate subsequently switches to an M2/Th2 polarization which plays a central role in the pathogenesis of fibrosis. We observed that abatacept led to a marked reduction of both inflammatory M1 and profibrotic M2 macrophage infiltrates. In addition, abatacept has been recently shown in inflammation-driven dermal fibrosis to regulate T-cell activation and infiltration, as well as the production of proinflammatory and profibrotic cytokines, leading to decreased resident fibroblast activation and reduced excessive collagen production [5]. Inhibition of activation and infiltration of T cells and macrophages may reduce cell proliferation and death [39], which is in line with decreased T-cell proliferation observed in lesional lungs of Fra-2 mice with abatacept treatment.

Our study has several limitations that deserve consideration. Liver involvement is a classical feature of preclinical and human cGvHD but is not usually affected in SSc. We used ALT/AST serum levels only to assess liver involvement, without complementary histologic evaluation. The merit of a cell-counting method on lesional tissue may appear less consistent than cell quantification performed by flow cytometry. However, we have developed a robust proficiency with this automated counting method, which was performed by two experienced independent investigators [5, 8, 19]. Lung compliance was not directly measured, but was indirectly reflected by the evaluation of FRC. It is also noteworthy that the impact of abatacept on survival in both mouse models has not been assessed.

## Conclusions

Taken together, our findings demonstrate how the inhibition of proinflammatory T-cell function, along with effects on macrophages (Additional file 5), yields significant therapeutic benefits in models of SSc organ damage. T-cell costimulation blockade might be therapeutically exploited to treat SSc patients with visceral organ involvement. The benefit of this strategy should be further compared with classical immunosuppressive approaches and emerging direct antifibrotic therapies. There is a strong rationale to evaluate T cell-targeting strategies in SSc given the importance of T cells in the early and inflammatory stages of the disease and accumulating preclinical evidence of a beneficial effects of targeted immunotherapy targeting T-cell costimulatory pathways, including OX40L, DNAM-1, or CTLA-4 [5, 8, 40]. In addition, a therapeutic approach for T cell-mediated diseases would target antigen-specific T cells involved in the disease, without leading to generalized immunosuppression. As a drug already in clinical use, and given its favorable safety profile in this study and in rheumatic patients, abatacept may be more translationally relevant than other means for targeting T cells currently being explored for the treatment of SSc.

## Additional files

Additional file 1: Abatacept prevents cGvHD-associated liver involvement. Serum alanine aminotransferase (ALT) (A) and serum aspartate aminotransferase (AST) (B) levels were substantially reduced in the serum of abatacept-treated cGvHD mice compared with control IgG1-treated cGvHD mice. A total of 32 mice were used (12 allogeneic control IgG1-treated mice, 12 abatacept 1 mg/mL-treated mice, and 8 control syngeneic BALB/c mice). Values are the median ± IQR. Statistics are from post-hoc Dunnett’s multiple comparison test. *P < 0.05, **P < 0.01. ALLO allogeneic, cGvHD chronic graft-versus-host disease, SYN syngeneic. (TIFF 294 kb)

Additional file 2: Abatacept alleviates gut involvement in experimental cGvHD. Representative images of inflammatory cell infiltration in lesional colon sections assessed by immunohistochemistry for CD45 (A). Increased submucosal CD45^+^ cell infiltration was detected in IgG1-treated cGvHD mice (A and B). CD45^+^ cell infiltration was markedly reduced in allogeneic cGvHD mice receiving abatacept (A and B). Representative images of cell death evaluation in lesional colon sections assessed by immunohistochemistry for Annexin-V (C). Cell death was prominent in IgG1-treated allogeneic cGvHD mice and was markedly reduced upon treatment with abatacept (C and D). A total of 32 mice were used (12 allogeneic control IgG1-treated mice, 12 abatacept 1 mg/mL-treated mice, and 8 control syngeneic BALB/c mice). Values are the median ± IQR. Statistics are from post-hoc Dunnett’s multiple comparison test. *P < 0.05, **P < 0.01, ****P < 0.0001. ALLO allogeneic, cGvHD chronic graft-versus-host disease, SYN syngeneic. (TIFF 6830 kb)

Additional file 3: Abatacept alleviates lung fibrosis in the Fra-2 mouse model. Representative images of second harmonic generation (SHG) performed to evaluate the accumulation of fibrillar collagen (A). SHG showed fibrillar collagen in Fra-2 mice treated with IgG1 (in pink), but not in mice receiving abatacept 10 mg/mL (A and B). Scale bar = 50 μm. Second harmonic scores were higher in Fra-2 mice receiving IgG1 or abatacept 1 mg/mL compared with Fra-2 mice treated by abatacept 10 mg/mL. A total of 27 mice were used (5 C57BL/6 mice, 6 Fra-2 control IgG1, 8 Fra-2 abatacept 1 mg/mL, and 8 Fra-2 abatacept 10 mg/mL). Values are the median ± IQR. Statistics are from post-hoc Dunnett’s multiple comparison test. *P < 0.05. (TIFF 5250 kb)

Additional file 4: Abatacept decreases levels of fibrogenic markers in lesional lungs of Fra-2 transgenic mice. Protein levels of MCP1 (A) and osteopontin (OPN) (B) were markedly reduced on treatment with abatacept 10 mg/mL. Protein levels of TGF-β (C) were significantly reduced on treatment with 1 mg/mL and 10 mg/mL abatacept compared with control IgG1-treated mice in lesional lungs of Fra-2 transgenic mice. A trend was observed for decreased concentrations of TIMP1 (D) in abatacept-treated mice. A total of 27 mice were used (5 C57BL/6 mice, 6 Fra-2 control IgG1, 8 Fra-2 abatacept 1 mg/mL, and 8 Fra-2 abatacept 10 mg/mL). Values are the median ± IQR. Statistics are from post-hoc Dunnett’s multiple comparison test. *P < 0.05. (TIFF 499 kb)

Additional file 5: Abatacept alleviates inflammation-driven fibrosis by suppressing the immune response. Schematic cartoon of the mechanism of action of abatacept in systemic sclerosis (SSc) based on results presented in this study and previously published results in skin fibrosis [5]. In the early stages of SSc, T cells are activated through their TCR and receive costimulation via CD28 from CD80/CD86-expressing antigen-presenting cells. The full activation of T cells enhances the activation of dermal/lung fibroblasts through the action of proinflammatory and/or profibrotic cytokines (IL-6, IL-10). This also involves the proinflammatory action of macrophages. During abatacept treatment, the drug blocks CD80/CD86-mediated costimulation by macrophages and B cells, leading to inhibition of T-cell activation, proliferation, and/or infiltration. The effects on macrophages lead to lower maturation and infiltration. B-cell infiltration is also reduced. As a consequence of the effect on T cells, B cells, and macrophages, the progression of dermal, lung, and vessel fibrosis is blocked. (TIFF 1546 kb)

## Abbreviations

ALT: Alanine aminotransferase
AST: Aspartate aminotransferase
cGvHD: Chronic graft-versus-host disease
CT: Computed tomography
CTLA: Cytotoxic T lymphocyte-associated molecule
FRC: Functional residual capacity
ILD: Interstitial lung disease
MCP: Monocyte chemoattractant protein
OPN: Osteopontin
PH: Pulmonary hypertension
RVH: Right ventricular hypertrophy
RVSP: Right ventricular systolic pressure
SHG: Second harmonic generation
SSc: Systemic sclerosis
TGF: Transforming growth factor
TPEF: Two-photon excited fluorescence
