# Supplementary figures and images for: T-cell costimulation blockade is effective in experimental digestive and lung tissue fibrosis
Source: Arthritis Res Ther. 2018 Aug 29;20:197. doi: 10.1186/s13075-018-1694-9 (PMC6116494; doi:10.1186/s13075-018-1694-9)

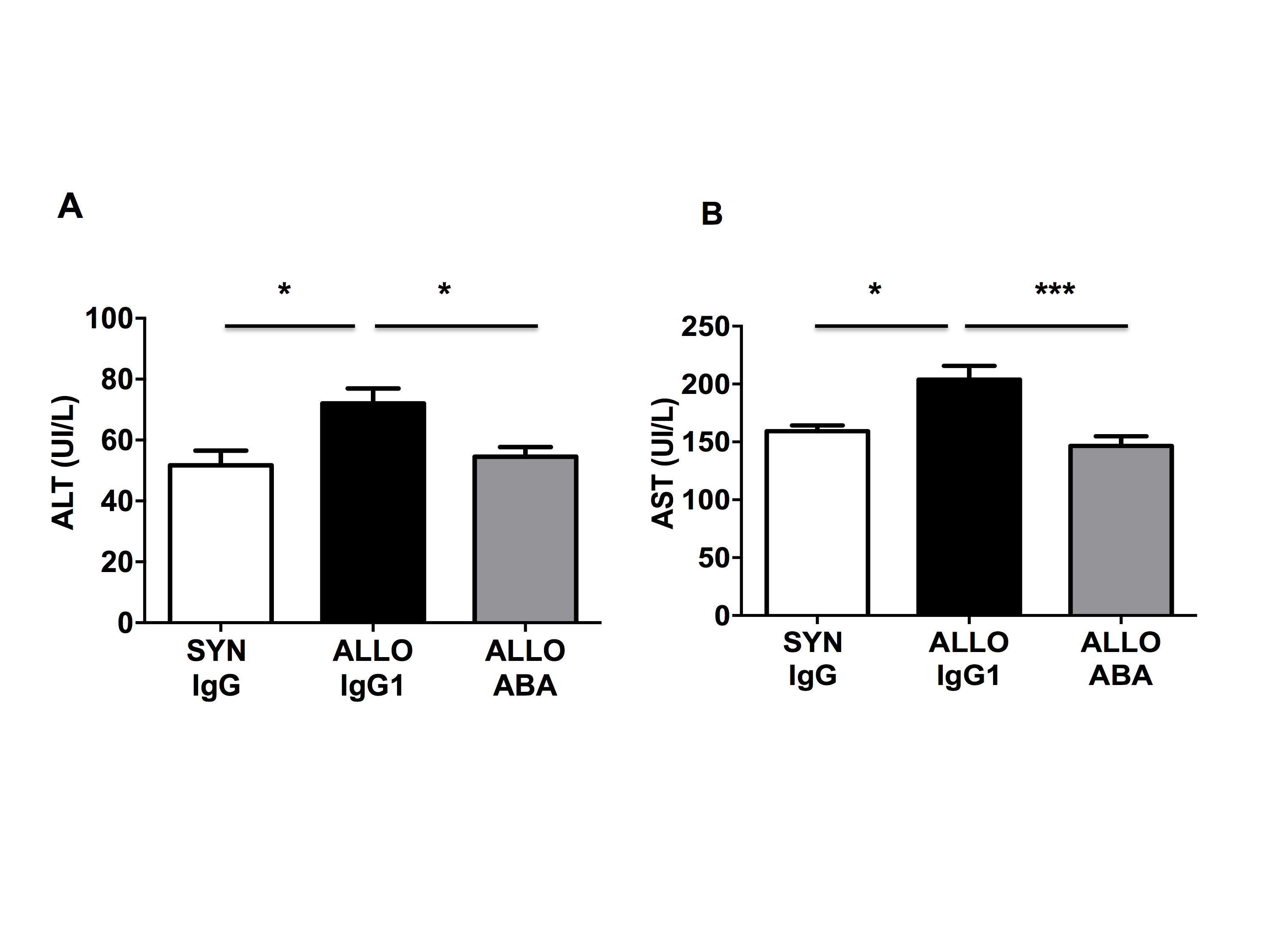

Supplement: Supplementary file 1 — Abatacept prevents cGvHD-associated liver involvement. Serum alanine aminotransferase (ALT) (A) and serum aspartate aminotransferase (AST) (B) levels were substantially reduced in the serum of abatacept-treated cGvHD mice compared with control IgG1-treated cGvHD mice. A total of 32 mice were used (12 allogeneic control IgG1-treated mice, 12 abatacept 1 mg/mL-treated mice, and 8 control syngeneic BALB/c mice). Values are the median ± IQR. Statistics are from post-hoc Dunnett’s multiple comparison test. *P < 0.05, **P < 0.01. ALLO allogeneic, cGvHD chronic graft-versus-host disease, SYN syngeneic. (TIFF 294 kb) [file 13075_2018_1694_MOESM1_ESM.tiff]

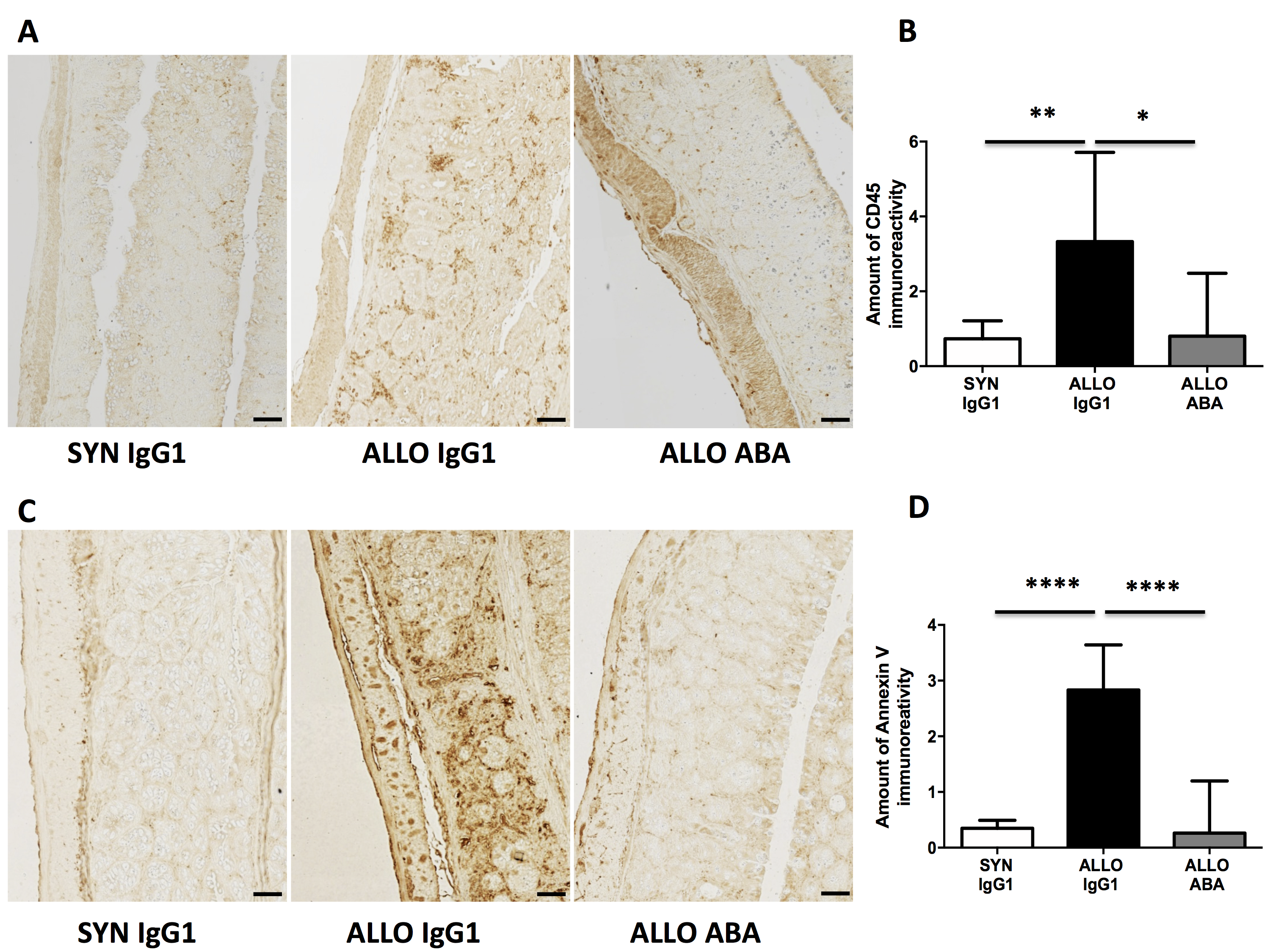

Supplement: Supplementary file 2 — Abatacept alleviates gut involvement in experimental cGvHD. Representative images of inflammatory cell infiltration in lesional colon sections assessed by immunohistochemistry for CD45 (A). Increased submucosal CD45+ cell infiltration was detected in IgG1-treated cGvHD mice (A and B). CD45+ cell infiltration was markedly reduced in allogeneic cGvHD mice receiving abatacept (A and B). Representative images of cell death evaluation in lesional colon sections assessed by immunohistochemistry for Annexin-V (C). Cell death was prominent in IgG1-treated allogeneic cGvHD mice and was markedly reduced upon treatment with abatacept (C and D). A total of 32 mice were used (12 allogeneic control IgG1-treated mice, 12 abatacept 1 mg/mL-treated mice, and 8 control syngeneic BALB/c mice). Values are the median ± IQR. Statistics are from post-hoc Dunnett’s multiple comparison test. *P < 0.05, **P < 0.01, ****P < 0.0001. ALLO allogeneic, cGvHD chronic graft-versus-host disease, SYN syngeneic. (TIFF 6830 kb) [file 13075_2018_1694_MOESM2_ESM.tiff]

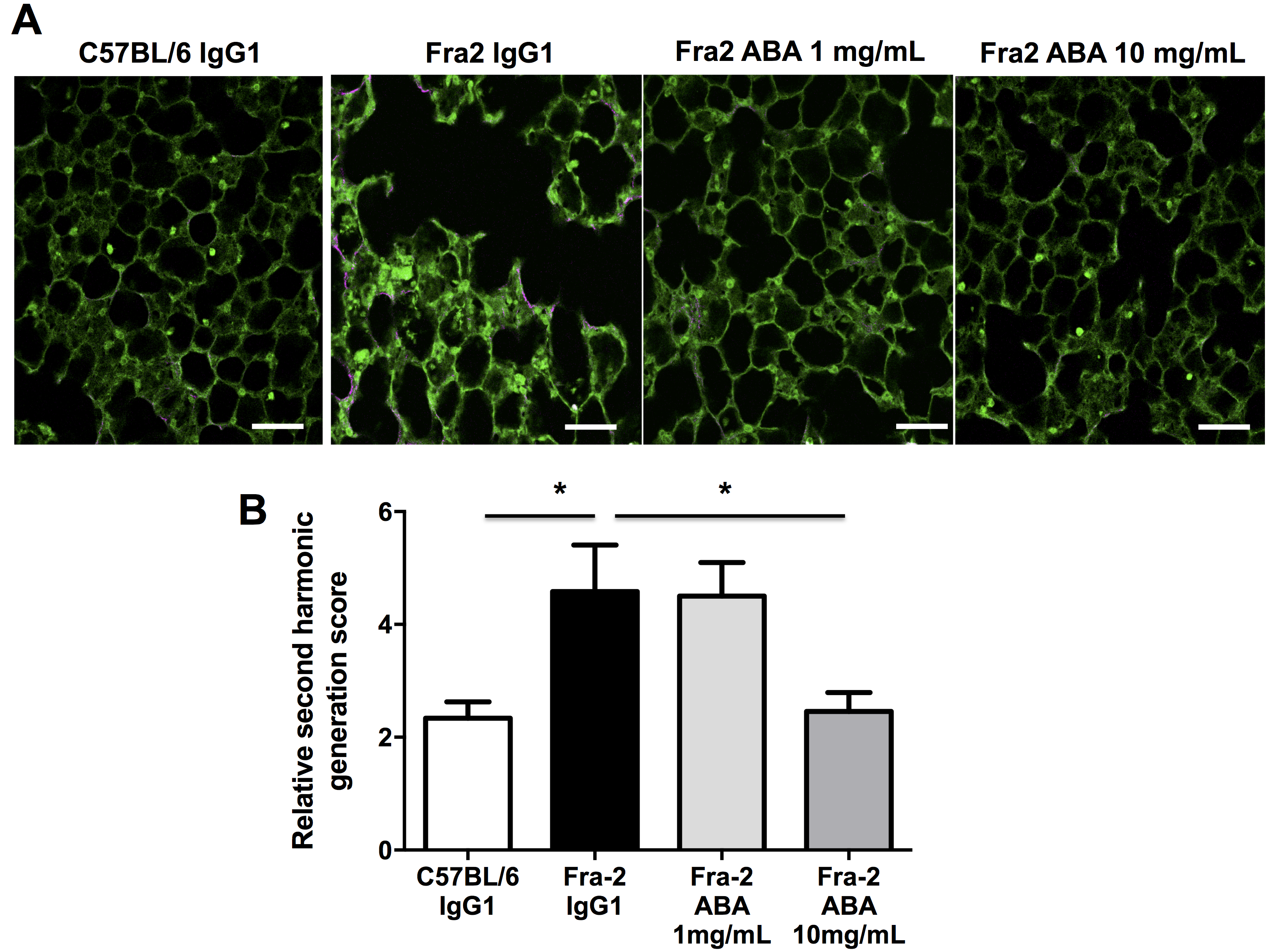

Supplement: Supplementary file 3 — Abatacept alleviates lung fibrosis in the Fra-2 mouse model. Representative images of second harmonic generation (SHG) performed to evaluate the accumulation of fibrillar collagen (A). SHG showed fibrillar collagen in Fra-2 mice treated with IgG1 (in pink), but not in mice receiving abatacept 10 mg/mL (A and B). Scale bar = 50 μm. Second harmonic scores were higher in Fra-2 mice receiving IgG1 or abatacept 1 mg/mL compared with Fra-2 mice treated by abatacept 10 mg/mL. A total of 27 mice were used (5 C57BL/6 mice, 6 Fra-2 control IgG1, 8 Fra-2 abatacept 1 mg/mL, and 8 Fra-2 abatacept 10 mg/mL). Values are the median ± IQR. Statistics are from post-hoc Dunnett’s multiple comparison test. *P < 0.05. (TIFF 5250 kb) [file 13075_2018_1694_MOESM3_ESM.tiff]

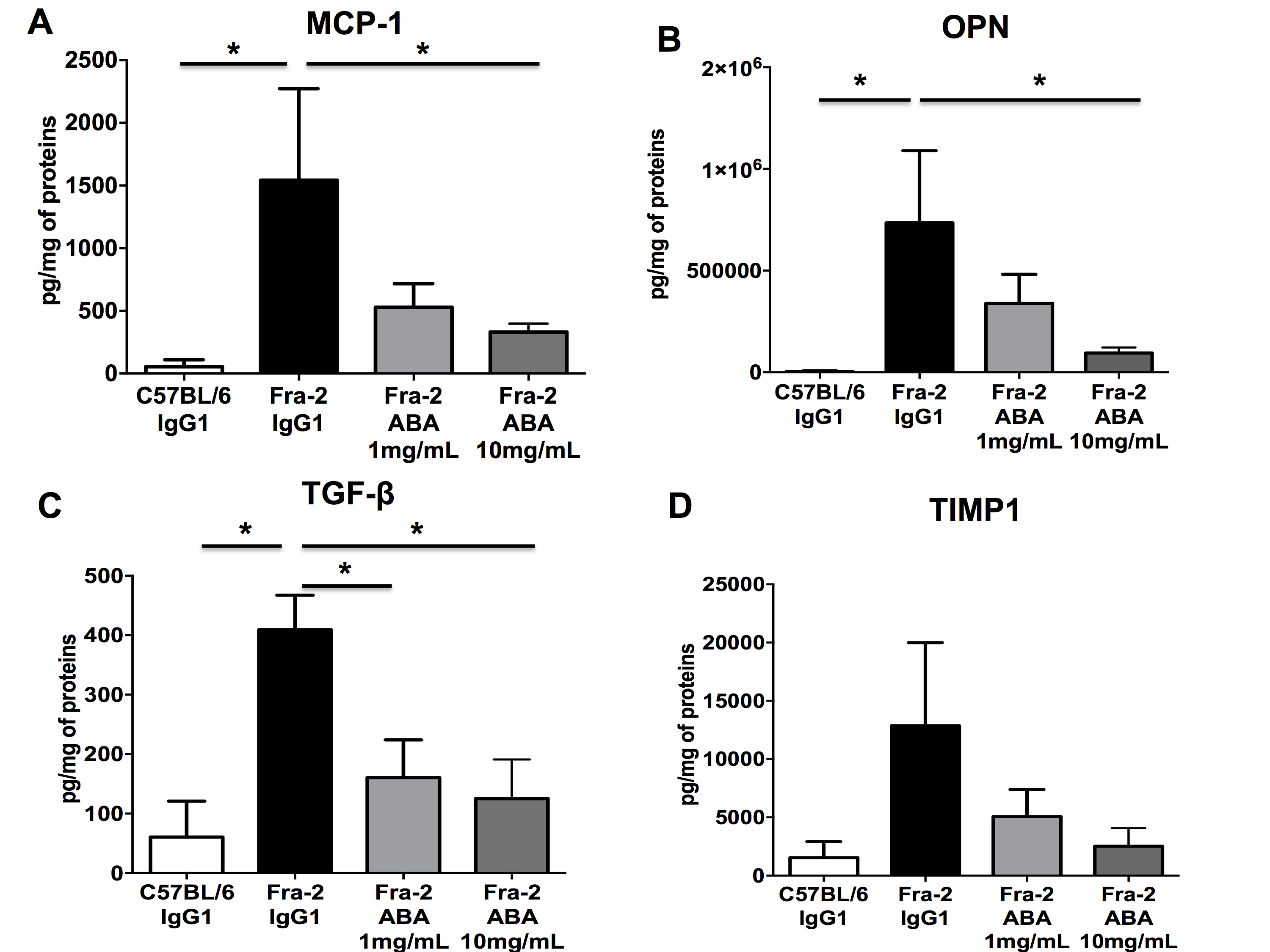

Supplement: Supplementary file 4 — Abatacept decreases levels of fibrogenic markers in lesional lungs of Fra-2 transgenic mice. Protein levels of MCP1 (A) and osteopontin (OPN) (B) were markedly reduced on treatment with abatacept 10 mg/mL. Protein levels of TGF-β (C) were significantly reduced on treatment with 1 mg/mL and 10 mg/mL abatacept compared with control IgG1-treated mice in lesional lungs of Fra-2 transgenic mice. A trend was observed for decreased concentrations of TIMP1 (D) in abatacept-treated mice. A total of 27 mice were used (5 C57BL/6 mice, 6 Fra-2 control IgG1, 8 Fra-2 abatacept 1 mg/mL, and 8 Fra-2 abatacept 10 mg/mL). Values are the median ± IQR. Statistics are from post-hoc Dunnett’s multiple comparison test. *P < 0.05. (TIFF 499 kb) [file 13075_2018_1694_MOESM4_ESM.tiff]

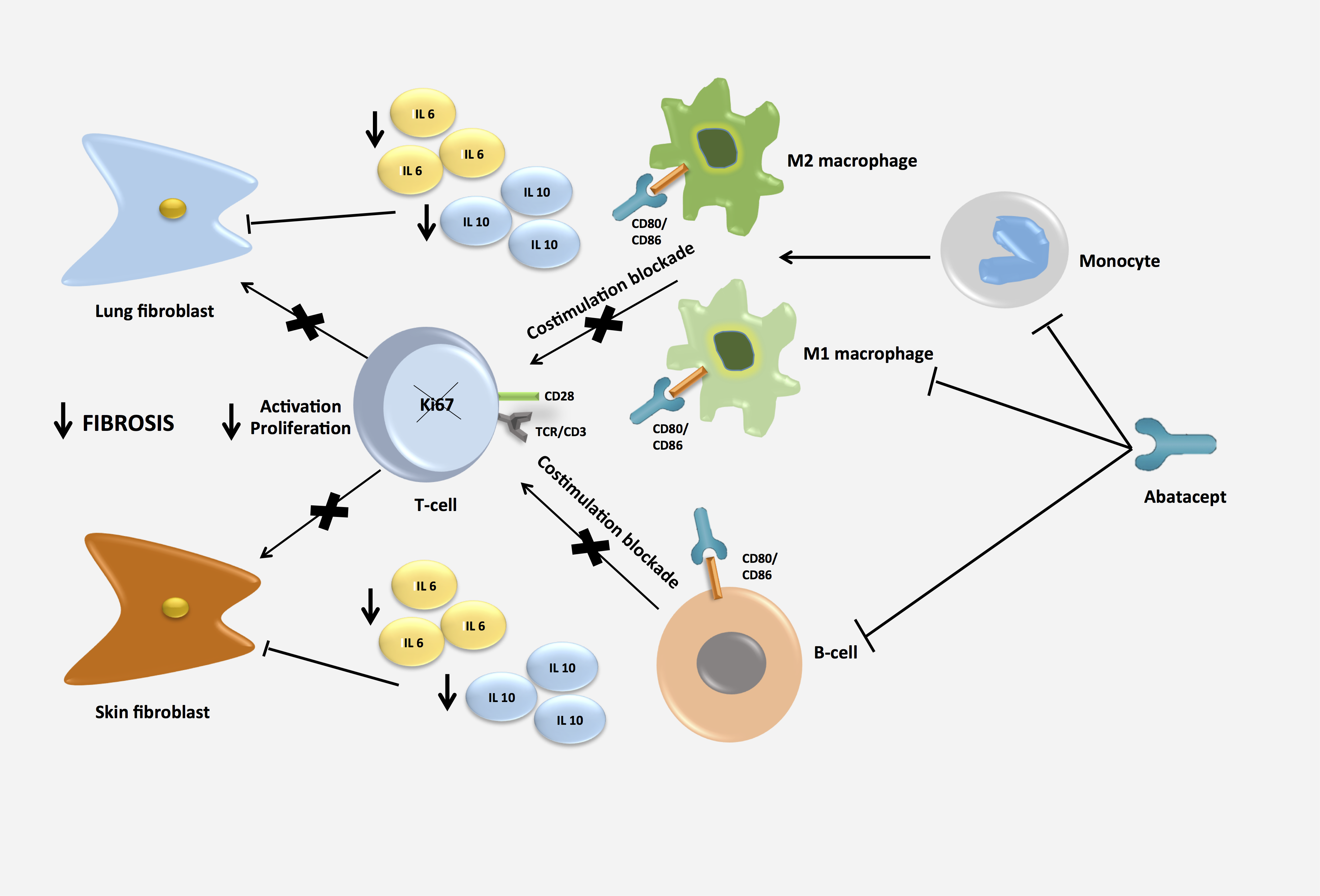

Supplement: Supplementary file 5 — Abatacept alleviates inflammation-driven fibrosis by suppressing the immune response. Schematic cartoon of the mechanism of action of abatacept in systemic sclerosis (SSc) based on results presented in this study and previously published results in skin fibrosis [5]. In the early stages of SSc, T cells are activated through their TCR and receive costimulation via CD28 from CD80/CD86-expressing antigen-presenting cells. The full activation of T cells enhances the activation of dermal/lung fibroblasts through the action of proinflammatory and/or profibrotic cytokines (IL-6, IL-10). This also involves the proinflammatory action of macrophages. During abatacept treatment, the drug blocks CD80/CD86-mediated costimulation by macrophages and B cells, leading to inhibition of T-cell activation, proliferation, and/or infiltration. The effects on macrophages lead to lower maturation and infiltration. B-cell infiltration is also reduced. As a consequence of the effect on T cells, B cells, and macrophages, the progression of dermal, lung, and vessel fibrosis is blocked. (TIFF 1546 kb) [file 13075_2018_1694_MOESM5_ESM.tiff]
